# Intravenous Thrombolysis for Acute Ischemic Stroke in Patients With Cardiac Myxoma: A Case Series and Pooled Analysis

**DOI:** 10.3389/fneur.2022.893807

**Published:** 2022-05-12

**Authors:** Jie Rao, Zi Tao, Qiongqiong Bao, Mingxia Jiang, Enyang Zhou, Xueli Cai, Fangwang Fu

**Affiliations:** ^1^Department of Neurology, The Second Affiliated Hospital and Yuying Children's Hospital of Wenzhou Medical University, Wenzhou, China; ^2^Department of Neurology, The Fifth Affiliated Hospital of Wenzhou Medical University, Lishui, China; ^3^Department of Neurology, Affiliated Yueqing Hospital, Wenzhou Medical University, Wenzhou, China; ^4^Department of Rehabilitation, The First Affiliated Hospital of Wenzhou Medical University, Wenzhou, China; ^5^Department of Neurology, Qingtian People's Hospital, Lishui, China

**Keywords:** stroke, myxoma, intravenous thrombolysis, bridging therapy, alteplase, efficacy

## Abstract

**Background and Purpose:**

Acute ischemic stroke (AIS) is a major life-threatening consequence of cardiac myxoma (CM) and leads to a poor prognosis. Although intravenous thrombolysis (IVT) is the first-line treatment for AIS, its efficacy and safety in CM-AIS have not been established. Currently, there are only limited data from case reports. Our study aimed to investigate the clinical characteristics of CM-AIS and evaluate the safety and efficacy of IVT for CM-AIS patients.

**Methods:**

Fourteen CM-AIS patients who received IVT between January 2016 and December 2021 were identified from our multicenter stroke registry databases. Clinical, neuroimaging and outcome data were analyzed. We then performed a pooled analysis of the published literature from inception to December 2021.

**Results:**

Of the 14 CM-AIS patients, nine were treated with IVT alone, and five were treated with bridging therapy (BT). The median age was 51.5 years, and 57.1% were female. The median onset-to-needle time was 160 min. The median National Institute of Health Stroke Score (NIHSS) decreased from 15.5 at presentation to 13 24 h after IVT. Very early neurological improvement (VENI) was observed in one patient. Hemorrhagic transformation (HT) was observed in five (35.7%) patients, and only one patient was symptomatic (7.1%). Three-month favorable outcomes were achieved in six patients (66.7%) who underwent IVT alone and three patients (60%) who received BT, which resulted in a total proportion of favorable outcomes of 64.3%. None of the patients died at 3 months follow-up. Forty-seven cases (15 BT patients) were included for the pooled analysis. The median NIHSS score was 16.5, and VENI was observed in 10 (21.3%) patients. HT was detected in 11 patients (23.4%), and four (8.5%) patients were symptomatic. Favorable outcomes at 3 months were achieved in 61.7% of patients, 56.3% of patients who underwent IVT alone, and 73.3% of patients who received BT. The 3-month mortality rate was 4.3%.

**Conclusions:**

IVT is a potentially safe and efficient treatment for CM-AIS patients. Further studies with larger sample sizes are required to provide more evidence on the safety and efficacy of IVT and BT in CM-AIS patients.

## Introduction

Cardiac myxoma (CM) is one of the most common primary cardiac tumors. A prominent presenting feature and major life-threatening consequence of CM is embolism (1), which has a prevalence of up to 50% in CM patients. Moreover, the cerebrovascular system is most commonly involved and affects more than half of all embolic events (2, 3). CM-related ischemic stroke only accounts for 0.5% of all strokes. However, nearly 30% of CM patients present with CM-related ischemic stroke (3, 4). Its rarity presents practical challenges for prospective studies and clinical randomized controlled trials (RCTs) on CM-related ischemic stroke patients. There is limited low-grade evidence on the therapeutic strategy for CM-related ischemic stroke, with most arising from case reports or small case series. Intravenous thrombolysis (IVT) with recombinant tissue plasminogen activator is the mainstay of reperfusion treatment for most acute ischemic stroke (AIS) patients and has been tried in several case reports of CM with limited success. In addition, the theoretical increase in cerebral hemorrhage risk, which is based on potential CM-related cerebral aneurysms and microbleeds, initially contraindicated CM patients to thrombolytic therapy. Conflicting evidence on the efficacy and safety of IVT in CM-related AIS (CM-AIS) has been reported in only a handful of case reports. Therefore, the AHA/ASA 2019 guidelines for AIS provide weak recommendations (Class IIb) for IVT in the management of CM-AIS (5). IVT “may be reasonable” for patients with severe disabling stroke based on individual risk–benefit assessments (5, 6). CM-related AIS is closely associated with a poor prognosis; thus, evaluating the efficacy and safety of IVT for patients with CM-AIS is crucial.

Given the clinical uncertainties related to IVT treatment in patients with CM-AIS, the present study aimed to elucidate the safety, efficacy, and clinical outcomes of IVT in patients with CM-AIS. To the best of our knowledge, this is the largest case series reporting the use of IVT in patients with CM-AIS. Additionally, we reviewed all previous case studies of patients who received IVT.

## Materials and Methods

### Study Design

This study was comprised of two parts: the first part reported on 14 cases from our multicenter stroke registry databases, and the second part undertook a comprehensive literature review. The study protocol was approved by local institutional review boards. The study was a retrospective review of electronic medical records, and informed consent for publication was obtained from all patients, in accordance with the Declaration of Helsinki (7).

### Retrospective Multicenter Case Series

#### Patient Selection

A retrospective review of all consecutive AIS patients who received IVT within 4.5 h of AIS onset from January 2016 to December 2021 was performed using stroke registry databases of seven comprehensive stroke centers in Zhejiang, Southeastern China: Qingtian People's Hospital, Ningbo Second Hospital, Huangyan Hospital, the Affiliated Yueqing Hospital, the First, Second, and Fifth Affiliated Hospitals of Wenzhou Medical University. Because of the relative rarity of CM-AIS, all patients who had CM that was identified either before or after surgery were enrolled in the study, regardless of whether they received bridging therapy (BT).

#### Clinical Data

Clinical data were collected from the electronic medical records system by two neurologists who were blind to participant conditions. Demographic characteristics and clinical features, which included risk factors, medical history, medication history, clinical presentation, National Institutes of Health Stroke Scale (NIHSS) score, and modified Rankin Scale (mRS) score (before the stroke, at discharge, and 3 months after onset), were collected. We also collected laboratory data, which included blood routine tests, blood lipids, glycosylated hemoglobin, and homocysteine coagulation profiles. MRS score was assessed at discharge and the 3-month follow-up by trained neurologists. Relapse of embolic events was also recorded at the 3-month follow-up.

#### Reperfusion Procedures

All therapeutic modalities and medical judgments were performed in accordance with the institutional and international guidelines for the early management of AIS (5). IVT treatment was administered as soon as possible if patients fulfilled the criteria for IVT within 4.5 h of stroke onset. The attending stroke neurologists decided whether BT was required according to patients' baseline patency of cerebral vessels and clinical condition on the second visit during the IVT procedure. NIHSS score was re-assessed 1 h after the initiation of IVT (or at the end of IVT). Very early neurological improvement (VENI) was evaluated according to a previously described (8) definition of an improvement of more than 4 points from baseline or an NIHSS score of 0 or 1.

Alteplase dose, onset-to-door time (OTD), door-to-needle time (DTN), onset-to-needle time (OTN), and complications of IVT data were obtained from medical records. Procedure parameters of BT were also collected if performed.

#### Imaging Evaluation

All imaging data were obtained from the image records system and analyzed by stroke neuroradiologists blinded to clinical and outcome data. Baseline brain computed tomography (CT) was performed at admission and analyzed using the Alberta Stroke Program Early CT Score (ASPECTS). Follow-up CT was performed 24 h after IVT and repeated when necessary. Brain magnetic resonance imaging (MRI) was undertaken within 48 h of stroke ictus if the condition of the patient allowed. Hemorrhagic transformation (HT) was assessed *via* MRI. If the patient was unable to tolerate the MRI scan, HT was assessed *via* CT 24 h after IVT and categorized according to the criteria of the European Cooperative Acute Stroke Study II (ECASS-II) into different radiologic subtypes: hemorrhagic infarction (HI) and parenchymal hemorrhage (PH). Symptomatic intracerebral hemorrhage (ICH) was defined as any ICH with an increase of more than 4 points on the NIHSS or clinical deterioration (9).

#### Evaluation of CM

All patients were routinely screened for cardiogenic embolism using transthoracic echocardiography (TTE) and Holer electrocardiogram. If TTE failed to detect CM, transesophageal echocardiography (TEE) was carried out in patients strongly suspected of having a cardioembolic stroke. Usually, myxoma can be identified easily using echocardiography. A cardiac MRI was performed if the patient showed normal echocardiography but had a histopathologically retrieved embolus diagnosed as a myxoma fragment. The location, tumor surface, and maximum tumor diameter of CM were obtained from the imaging database. Possible clinical manifestations of CM were screened using medical records, and a medical history questionnaire was administered over the telephone. Surgery-specific information, surgical complications, and pathological information of CM were reviewed if available.

#### Neurological Outcomes

Our primary outcome was the 3-month mRS score. For patients lost to follow-up, the mRS score at discharge was used instead. A favorable functional outcome was defined as an mRS score change of fewer than 2 points from the premorbid state to 3 months. Safety outcomes included death 3 months after AIS and the development of ICH or symptomatic ICH (sICH).

### Systematic Review and Pooled Analysis

**Figure 1:**
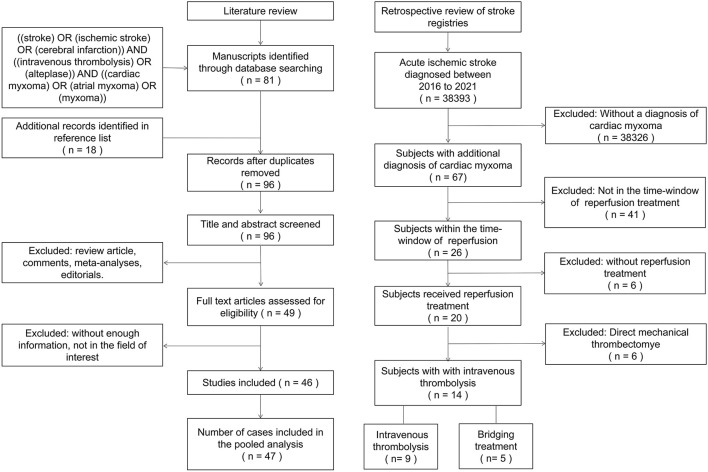
Literature review and retrospective review flow-charts.

A systematic review of the published literature was conducted by searching PubMed and CNKI databases according to PRISMA guidelines on December 30th, 2021 (a flow diagram is provided in Figure 1). The search strategy was: (“stroke” OR “ischemic stroke” OR “cerebral infarction”) AND (“intravenous thrombolysis” OR “alteplase”) AND (“cardiac myxoma” OR “atrial myxoma” OR “myxoma”). Additionally, references of identified publications were manually searched to identify relevant publications that were not captured by the initial search. We imposed no language or publication date restrictions for the search. Studies using any design were eligible for inclusion if they reported a case or case series of CM-AIS patients treated with IVT. Two authors independently screened the included articles and extracted data using a predefined standardized data collection form.

Descriptive statistics are presented as means (standard deviations), medians [interquartile ranges (IQRs)], and numbers (proportions). Our study was descriptive in nature and comprised very small sample sizes; therefore, we did not perform any formal statistical comparisons.

## Results

### Retrospective Multicenter Case Series

A total of 38,393 AIS patients were enrolled in the stroke registries during the study period. Of these, 67 patients (1.75‰) were identified as having CM-AIS. Twenty patients with CM-AIS were treated with reperfusion, of whom nine were treated with IVT alone, six with mechanical thrombectomy (MT) alone, and five with BT. As a result, we identified 14 CM-AIS patients treated with IVT. The flow chart of the patients selection is shown in Figure 1. Individual clinical details are described in Table 1, and the characteristics of the cohort are summarized in Table 2. The median age of patients was 51.5 years (range 38–75 years), and eight patients (57.1%) were women. Five patients (35.7%) had traditional stroke risk factors, of which hypertension (4 patients, 28.6%) was the most common. Three patients developed AIS despite prior antiplatelet treatment in two patients and prior anticoagulation treatment in one. The median NIHSS score at admission was 15.5 (range 5–28).

**Table 1:** Characteristics of patients with CM-AIS treated with IVT.

	**Age/ sex**	**Risk factor**	**Initial** **NIHSS**	**Multivessel territories involvement**	**Peripheral vascular involvement**	**ASPE-CTS**	**Intravenous thrombolysis**									**BT**	**Cardiac myxoma**						**Favorable** **outcome**
							**OTD** **(min)**	**OTN** **(min)**	**VENI**	**NIHSS** **at 24 h**	**HT**	**sICH**	**MBE**	**NIHSS** **on** **discharge**	**mRS** **at** **3 months**		**Site and** **diameter** **(mm)**	**Irregular** **surface**	**Prestroke symptom**	**Removal time**	**Drug** **before** **removal**	**Relapse** **before** **removal**	
1	59/F	No	28	Yes	Coronary artery	8	30	175	No	29	Yes, HI2	No	No	25	5	No	LA, 30	Yes	No	5 m	Warfarin	Myocardial infarction	No
2	66/F	No	15	No	No	10	100	135	No	12	No	No	No	11	3	No	LA, 12	Yes	No	1 y	Warfarin	Pulmonary embolism	No
3	62/M	Smoking	22	No	Left brachial artery	9	135	180	No	20	Yes, SAH and HI2	No	No	6	2	No	LA, 70	Yes	Syncope, dizziness	23 d	LMWH	No	Yes
4	48/F	No	22	Yes	No	10	160	210	No	14	No	No	No	6	1	No	LA, 16	Yes	No	59 d	Aspirin, clopidogrel	No	Yes
5	42/F	No	13	Yes	No	9	100	140	Yes	8	No	No	No	6	0	No	LA, 32	Yes	No	3 m	Rivaroxaban	No	Yes
6	75/F	HTN, CHD, AF	5	No	No	9	158	202	No	3	No	No	No	0	2	No	LA, 44	Yes	No	Not performed	Rivaroxaban	Cerebral infarction	Yes
7	55/M	No	12	Yes	No	9	127	162	No	6	Yes, HI2	No	No	3	1	No	LA, 60	Yes	No	20 d	LMWH	No	Yes
8	57/F	HTN, DM	16	No	No	9	112	160	No	22	No	No	Yes	12	4	No	LA, 40	Yes	No	6 m	LMWH	No	No
9	42/M	No	10	No	No	9	151	198	No	6	No	No	No	4	2	No	LA, 31	Yes	No	1 m	Aspirin, clopidogrel	No	Yes
10	38/F	No	11	No	No	10	125	144	No	0	No	No	No	0	0	Yes	LA, 51	Yes	No	14 d	Aspirin	No	Yes
11	50/F	HTN	22	No	No	10	120	159	No	20	No	No	No	20	4	Yes	LA, 48	No	No	Not performed	Dabigatran	No	No
12	51/M	No	25	No	Right posterior tibial artery	9	116	160	No	19	Yes, PH2	No	No	6	1	Yes	LA, 41	No	No	24 d	LMWH	No	Yes
13	52/M	HTN, DM	12	No	No	10	70	109	No	6	No	No	No	2	0	Yes	LA, 49	Yes	No	17 d	Aspirin	Cerebral infarction	Yes
14	51/M	No	26	Yes	Left posterior tibial artery	8	20	48	No	22	Yes, PH2	Yes	Yes	25	5	Yes	LA, 43	Yes	No	50 d	Aspirin	No	No

*ASPECTS, Alberta Stroke Program Early CT Score; BT, bridging therapy; CHD, coronary heart disease; DM, diabetes mellitus; F, female; HI, hemorrhagic infarction; HT, hemorrhagic transformation; HTN, hypertension; LA, left atrium; M, male; LMWH, low molecular weight heparin; MBE, malignant brain edema; MCA, middle cerebral artery; NIHSS, National Institutes of Health Stroke Scale; mRS, modified Rankin Scale; OTD, onset-to-door time; OTN, Onset-to-needle time; PH, parenchymal hemorrhage; SAH, subarachnoid hemorrhage; sICH, symptomatic intracerebral hemorrhage; TIA, transient ischemic attack; VENI, very early neurological improvement*.

**Table 2:** Clinical characteristics, periprocedural and outcome results of CM-AIS patients treated with IVT.

	**Institutional cases** **(*n* = 14)**	**Literature cases** **(*n* = 47)**
**Demographic characteristics**		
Median age, years (IQR)	51.5 (46.5–59.75)	52 (40–64)
Female sex, % (*n*)	57.1 (8)	42.2 (19)
**Clinical characteristics**		
Stroke risk factors, % (*n*)	35.7 (5)	36.2 (17)
Prior antithrombotic treatment, % (*n*)	21.4 (3)	4.4 (2)
Median initial NIHSS (IQR)	15.5 (11.75–22.75)	16.5 (11–20)
Median ASPECTS (IQR)	9 (9–10)	N.A.
Large vessel occlusion on onset, % (*n*)	57.1 (8)	64.9 (24)
High density sign of artery, % (*n*)	0	9.1 (4)
Multivessel territories involvement, % (*n*)	35.7 (5)	37.2 (16)
Peripheral vascular involvement, % (*n*)	28.6 (4)	14.9 (7)
Cerebral aneurysms, % (*n*)	0	2.9 (1)
**IVT characteristics**		
IVT with standard dose, % (*n*)	100 (14)	83 (39)
Onset to door time (IQR)	118 (92.5–139)	60 (35.5–120)
Onset to needle time (IQR)	160 (138.75–184.5)	140 (96.25–180)
Door to needle time (IQR)	42 (35–47.25)	60 (30–120)
Very early neurological improvement, % (*n*)	7.1 (1)	21.3 (10)
NIHSS at 24 h (IQR)	13 (6–20.5)	6 (3–16.5)
NIHSS at discharge (IQR)	6 (2.75–14)	6 (0–15)
Bridging therapy, % (*n*)	35.7 (5)	31.9 (15)
**Site of infarction, % (** ***n*** **)**		
Anterior circulation, % (*n*)	71.4 (10)	79.1 (34)
Posterior circulation, % (*n*)	0	7 (3)
Simultaneous involvement, % (*n*)	28.6 (4)	13.9 (6)
Bilateral hemispheres, % (*n*)	28.6 (4)	25.6 (11)
**Cardiac myxoma**		
Prestroke clinical presentation, % (*n*)	7.1 (1)	27.7 (13)
Median tumor diameter, mm (IQR)	42 (30.75–49.5)	45 (32–60)
Tumor in left atrium, % (*n*)	100 (14)	83 (39)
Irregular tumor surface, % (*n*)	85.7 (12)	71.4 (20)
**Pre-removal antithrombotic treatment, % (** ***n*** **)**		
Antiplatelet agents, % (*n*)	35.7 (5)	47.1 (8)
Anticoagulants, % (*n*)	64.3 (9)	47.1 (8)
Combination, %(*n*)	0	5.8 (1)
Pre-removal embolism, *n* (%)	28.6 (4)	14.9 (7)
**Outcome**		
Hemorrhagic transformation, % (*n*)	35.7 (5)	23.4 (11)
Symptomatic ICH, % (*n*)	7.1 (1)	8.5 (4)
Decompressive craniectomy, % (*n*)	14.3 (2)	6.4 (3)
Median mRS (IQR)	2 (0.75–4)	2 (1–3)
Favorable outcome (mRS 0–2), % (*n*)	64.3 (9)	61.7 (29)
Mortality, % (*n*)	0	4.3 (2)

*The percentages for subcategories are based on the patients who have related data*.

*ASPECTS, Alberta Stroke Programme Early CT Score; IVT, intravenous thrombolysis; NIHSS, National Institutes of Health Stroke Scale; mRS, modified Rankin Scale*.

All patients had an ASPECTS of more than 8 (median 9, range 8–10). Hyperdense artery signs were not reported in any patient. Large vessel occlusion (LVO) was found in eight patients, most of whom were located in the anterior circulation. Multiple cerebral vascular involvements were identified in five cases (35.7%), and peripheral vascular involvement was found in four patients. No cerebral aneurysms were found.

The procedural details are outlined in Table 1. All patients received IVT with standard-dose alteplase (0.9 mg/kg). The median OTD and OTN were 118 min (IQR 92.5–139) and 160 min (IQR 138.75–184.5), respectively. None of the patients developed adverse immunological reactions during or after IVT. The median NIHSS score 1 h after IVT initiation was 15.5 (IQR 9.5–22.75). VENI was observed in only one patient. The median NIHSS score 24 h after onset was 13 (IQR 6–20.5), with a median decrease in NIHSS score of 4 (IQR 2–6). Five (35.7%) patients received MT after IVT. Significant neurological improvement 24 h after onset (i.e., a decrease in NIHSS score of more than 4 points) was observed in four (44.4%) patients who received IVT alone and four patients (80%) who received BT. Of note, one patient who underwent IVT alone showed a rapid increase in NIHSS score at 24 h due to malignant cerebral edema. Four patients who received BT underwent successful recanalization, and the retrieved emboli were determined to be part of the myxoma.

Acute bilateral infarctions were found in four patients (28.6%). Simultaneous involvement of both the posterior and anterior circulations was identified in four patients (28.6%). HTs were found in five patients (35.7%), of whom two had HI2, one had HI2 and subarachnoid hemorrhage (SAH), and two had PH2. Most HTs were asymptomatic. Only one patient (7.1%) with PH2 had clinical deterioration and subsequent malignant cerebral edema, which led to severe disability (mRS score of 5). Digital subtraction angiography was performed in the patient with SAH; however, no myxomatous aneurysms were identified. Nine patients were given anticoagulants, whereas the remaining five patients received antiplatelet agents. In one patient, ICH was detected in the repeated CT, and antithrombotic therapy was stopped. Four out of the five patients with an HT received anticoagulants after IVT. Four patients experienced thromboembolic events despite prompt antithrombotic therapy. Two patients experienced cerebral infarction, one had a pulmonary embolism, and the remaining one had a myocardial infarction. Three of these patients underwent surgical resection of the CM. Notably, no clinical thromboembolic events occurred after the surgery in these patients.

All CMs were located in the left atrium. AIS was the initial manifestation of CM in 92.9% of patients. Only one patient experienced dizziness, left brachial artery embolism, and several episodes of syncope. CM was diagnosed before IVT in only two patient (14.3%). All CMs were identified *via* echocardiography. Eight CMs were identified by TTE, and six were identified by TEE. The median diameter of CMs on echocardiography was 42 mm (range 12–70 mm). Irregular tumor surface was found in 12 patients (85.7%) *via* echocardiography. A total of 12 patients underwent CM resection, and the papillary type was found in eight patients (66.7%). There were no perioperative complications. Five (41.7%) patients had thrombi overlaying the myxoma. All CMs were benign, and malignant cells were not found in any patient.

Progression to malignant brain edema occurred in two patients, and both underwent urgent decompressive craniectomy. There were no deaths within 3 months in our study population. The median NIHSS score at discharge was 6 (IQR 2.75–14), and the median mRS score at 3 months was 2 (IQR 0.75–4). Favorable outcomes at 90 days were achieved in six patients (66.7%) who underwent IVT alone and three patients (60%) who received BT, which resulted in a total proportion of favorable outcomes of 64.3%.

### Cases in the Pooled Analysis

Our literature review rendered 45 case reports and one small case series. A total of 47 patients were included in the pooled analysis. Detailed results of the cases from the literature review are presented in Supplementary Table 1. The median age was 52 years (rang 4–79 years), and 8.5% were minors. Only 17 patients (36.2%) were older than 60 years. There was a predominance of males at 57.8%, with a median NIHSS score of 16.5 (IQR 11–20) at admission. Most patients (83.0%) received a standard-dose (0.9 mg/kg) of alteplase. Eight patients received low-dose alteplase.

The median OTD and OTN were 60 min (IQR 35.5–120) and 140 min (IQR 96.25–180), respectively. Hyperdense artery signs were recorded in only four patients (8.5%). VENI was found in 10 (21.3%) patients. BT was performed in 15 patients (31.9%), of whom only one showed rapid improvement, and two experienced rapid neurological deterioration. Only 21 patients had a reported NIHSS score at 24 h after IVT, for which the median score was 6 (IQR 3–16.5).

Sixteen (37.2%) patients had cerebral infarcts in multiple vascular territories, and 27 patients (62.8%) had involvement of only one vascular territory. Acute bilateral infarctions were found in 11 patients (25.6%). Simultaneous involvement of both the posterior and anterior circulations was identified in six patients (13.9%). Only one patient was reported to have three small aneurysms. HT was observed in 11 patients (23.4%), of whom five had PH2, three had HI1, two had HI2, and one had no information on HT classification. Most HTs were asymptomatic; four (8.5%) patients were symptomatic. Information on antithrombotic therapy was only provided for 17 cases. Eight patients were treated with antiplatelet agents, eight with anticoagulants, and one with a combination of antiplatelet agents and anticoagulants. Recurrence of thromboembolic events was reported in seven patients (14.9%) before CM removal. No clinical thromboembolic events were reported after the surgery.

Most CMs were located in the left atrium (39, 83.0%); three were located in the left ventricle, three in the bilateral atrium, one in the aortic valve, and one in the right ventricle. The median diameter of CMs was 45 mm (IQR 32–60 mm). Twenty-eight patients had data regarding the surface of the CM. An irregular surface was found in 20 patients (71.4%). A total of 40 patients underwent complete resection of the CM, with a median time of CM removal of 7 days (IQR 4–20 days). No perioperative complications or recurrence of CM were reported.

Malignant brain edema occurred in four (8.5%) patients, and urgent decompressive craniectomy was carried out in three patients. There were two deaths with a reported mortality of 4.3%. One patient died after peripheral vascular and cardiac surgeries, and another died because of multiple systemic complications. The median mRS score at follow-up was 2 (IQR 1–3). Favorable outcomes at 90 days were achieved in 29 (61.7%) patients: 56.3% (18/32) of patients received IVT alone and 73.3% (11/15) of patients received BT.

## Discussion

The incidence of primary cardiac tumors is very low, with a reported incidence of 0.001–0.3% (1). CM, one of the great mimickers, is the most prevalent entity of primary cardiac tumors and accounts for more than 50% of benign cardiac tumors in patients older than 16 years (10). Most CMs originate from subendocardial mesenchymal cells, mainly in the left atrium, with only 18% of CMs being located in the right atrium. The clinical manifestation of CM is diverse, varying from incidental detection on health checkups in the asymptomatic population to sudden cardiac death (1–4). A classic triad of clinical presentations has been recognized, which includes valvular obstruction symptoms, constitutional symptoms, and systemic embolization (1–4). Embolic events are the most common manifestation, with a reported prevalence of up to 50% in CM patients (3). Cardioembolism strokes have been reported to account for more than half of all embolic events (1, 4, 11). Although CM accounts for <1% of AIS in the total population, it must be considered an important differential diagnosis in patients without evidence of a common AIS etiology, especially in young people and children. AIS has been demonstrated to be a major factor that contributes to a poor prognosis in patients with CM (2, 12). Given the high mortality and disability rates of CM-AIS, there is a pressing medical need to assess the efficacy and safety of reperfusion treatment for CM-AIS.

CM can occur at any age but is particularly common between the third and sixth decades of life (1, 2, 13). The median age of CM at presentation is 50 years, which is similar to that of our case series. Previous studies of CM have shown strong female preponderance at a ratio of approximately 2:1 (14). However, the proportion of females (1.33:1) was much lower in our case series. In fact, the pooled analysis showed male preponderance. Similar to our findings, a lower proportion of females (1–1.4:1) in CM patients with ischemic stroke or embolic complications has been reported in previous studies (14, 15). One possible reason for this bias is that the male sex is a major risk factor for stroke. The traditional risk factor of cerebrovascular disease is more frequent in male patients than in female patients. CM patients with risk factors such as atrial fibrillation or hypertension are likely to have a high risk of embolism. Furthermore, several other risk factors of embolism have been reported, although results are inconsistent (16). For example, one study reported that small tumor size is independently associated with the risk of embolism (17), whereas other studies have presented opposing conclusions (18) or no association between tumor size and embolism risk (16). Other reported risk factors include tumor type and irregular surface (12, 19, 20). CHA_2_DS_2_-VASc scoring is recommended for predicting the risk of embolism in CM patients (19). A recent systematic review and meta-analysis focusing on risk factors of embolism in CM patients revealed that only hypertension, increased fibrinogen, irregular tumor surface, narrow tumor base, and atypical location were independent risk factors of embolism (16).

Because of the predominance of left-atrium CM, neurological complications are relatively common, and some can be devastating or even life-threatening. Neurological manifestations are reported to occur in 12–45% of CM patients (21). Ischemic stroke is the most common neurological manifestation and also the most common initial presentation of CM, at approximately 80% of reported cases (21). These observations are highly consistent with our data. AIS was the most common initial manifestation, affecting 92.9% of our cases. Although intracardiac obstruction, non-specific constitutional symptoms, and peripheral embolism are common occurrences in CM patients (2–4), very few of our CM-AIS patients experienced these symptoms. This may be attributed to the fact that patients with AIS tend to be younger and more likely to be male than those without AIS. When AIS is the main presentation of CM with embolic potential, the time to diagnosis will be much shorter. Moreover, the embolic potential generally originates from papillary type CM pathology, which usually has high mobility and an irregular surface (22). Thus, blockage and constitutional symptoms are relatively rare in CM-AIS patients. As a result, CM is often misdiagnosed even after the occurrence of AIS. The considerably lower prevalence of systemic presentations in our case series and pooled analysis than that of previous reports may be attributed to the design limitations of the study, which include the retrospective nature and potential for selection bias. Furthermore, minor and non-specific symptoms may not be included in medical records. In our study, CM was rarely diagnosed before AIS onset. CM diagnosis that preceded AIS was only observed in two (14.3%) patients in our case series and four (8.5%) patients in the pooled analysis. This result is in line with a previous comprehensive literature review that included 133 CM-related stroke patients (15). Although bedside TTE is strongly recommended in AIS, the decision to provide reperfusion treatment should not be delayed by the work-up of CM patients.

IVT remains the first-line reperfusion treatment for AIS (5). However, its safety and efficacy have not yet been established for CM-AIS patients. The 2019 AHA/ASA guidelines for the early management of AIS recommend that IVT is “reasonable” in CM-AIS patients who are likely to be severely disabled (5). Myxomatous aneurysms and metastatic lesions in the central nervous system are reported reasonably frequently (14, 22, 23). The reported prevalence of myxomatous aneurysms is 0–67% (11, 14, 23, 24), whereas metastatic lesions are found in 4.3% of patients with CM (14). However, the true incidence remains uncertain because all data are derived from small case series. Theoretically, myxomatous aneurysms are closely associated with high bleeding risk. Thus, CM was initially considered a relative contraindication to IVT. The pathophysiological mechanism underlying myxomatous aneurysms are currently unclear, although commonly accepted theories are the neoplastic process theory and micro-embolic damage theory (25, 26). To date, there have only been approximately 60 reported cases of myxomatous aneurysms (26). Because of its rarity, little is known about the nature and management of myxomatous aneurysms. In addition, the prevalence of hemorrhagic complications of aneurysm rupture has also been reported to be higher than that of other intracranial aneurysms, although the findings are inconsistent (25, 26). In a recent systematic review of 41 cases with myxomatous aneurysms, one-quarter of patients had intracranial hemorrhage due to aneurysm rupture (25). Another systematic review of 37 cases highlighted that conservative management with angiographic follow-up was reasonable given the evidence that most (75.9%) myxomatous aneurysms remain stable or regress during follow-up (26). The actual rupture risk of myxomatous aneurysms cannot be extrapolated on account of the rarity of the disease, especially in patients who undergo IVT. A recent study, which comprised 412 AIS patients with unruptured intracranial aneurysms, concluded that thrombolytic therapy did not increase the risk of ICH or sICH (27). However, this result cannot be directly applied to the clinical decision-making of CM-AIS because of the difference in pathological mechanisms of myxomatous aneurysms. As mentioned earlier, the time to diagnosis of CM is significantly shorter in patients who initially present with AIS. The presence of myxomatous aneurysms may be much less. To the best of our knowledge, among the CM-AIS patients treated with IVT, there has only been one patient reported to have aneurysms.

A literature review of thrombolytic therapy for CM-AIS described the safety and efficacy features in 23 cases (4 patients received intra-arterial thrombolysis, 19 patients underwent IVT) with this rare condition (28). Clinical improvement, which was defined as marked improvements in neurological symptoms or a reduction of NIHSS points at 4 or more than 4, was used as the main indicator to evaluate the efficacy of thrombolytic therapy in this study. It was achieved in 52.2% of the patients in this review, whereas a similar improvement rate (64.3%) was found in our multicenter series. These results were consistent with the previous studies of IVT (29, 30). For example, the rate of clinical improvement was 57.8% in the TIMS-China trial and the Shanghai Stroke Service System registry (29). The function outcome was not evaluated in Acampa's study, we extracted the data from this study and calculated a favorable outcome (mRS score ≤ 2) rate of 57.1% (28). Consistent with Acampa's study, favorable functional outcomes were achieved using IVT alone in six (66.7%) patients in our case series and 56.3% of patients in the pooled analysis. The above evidence indicates that IVT is similarly efficacious in CM-AIS patients. It is interesting that IVT is more effective in CM-AIS patients than in patients with atrial fibrillation (ranging from 32.2 to 52.6% across studies) (31). Although Acampa and colleagues reported a prevalence of ICH after thrombolytic therapy at 21.7%, which appeared lower than our case series (35.7%), the patients who received IVT had a similar prevalence of ICH (26.3%) was similar to our case series. Of these patients with hemorrhage complications, no major clinical deterioration or death was observed following hemorrhage. The reported prevalence of ICH was similar to that reported in the ECASS II (48.4%) (9) and ECASS III (27.0%) studies (30). Thus, the authors concluded that IVT is safe and effective for treating patients with CM-AIS (28). In contrast, a recent study involving nine CM-AIS patients with LVO reported no clinical improvement in patients after IVT (32). However, given the designs of these studies, publication and selective reporting biases were inevitable, which likely led to unreliable results. Thus, we conducted a multicenter retrospective study in patients who were consecutively enrolled, which minimized bias. Therefore, the estimates of our study are likely more in line with the actual situation. The sICH rates of our case series and pooled analysis were 7.1 and 8.5%, respectively, which are consistent with those of IVT studies in patients with atrial fibrillation (ranging from 4.0 to 18.2% across studies), the most common etiology of cardioembolism stroke (31, 33). Similar results have also been reported for the prevalence of HT (31). These results indicate that, to some extent, IVT is a safe intervention for CM-AIS patients with similar sICH and HT rates to patients with atrial fibrillation. Taken together, IVT may be effective and tolerable for CM-AIS patients, and IVT should not be withheld if CM-AIS is highly suspected. Only when AIS secondary to infective endocarditis cannot be excluded should IVT be critically discussed because of the significantly higher rate of sICH (34). Cerebral angiography using CTA or MRA is recommended prior to IVT for cerebral vessel evaluation, bleeding risk stratification, and minimizing the risk of ICH (11, 35).

The Enhanced Control of Hypertension and Thrombolysis Stroke Study (ENCHANTED) found that low-dose intravenous alteplase could reduce the risk of sICH which was the most worrisome complication of IVT (36). However, controversy persists over the patients who may benefit from low-dose alteplase. A subgroup analysis of ENCHANTED showed that low-dose alteplase had not any advantages over standard-dose alteplase for patients with lacunar AIS, even an increased the risk of ICH and sICH (36). Only for patients with moderate stroke, low-dose alteplase may associate with sICH reduction and non-inferior performance in efficacy (29). A nationwide study in Taiwan showed that low-dose alteplase posed a significantly increased risk of sICH in patients with atrial fibrillation than standard-dose alteplase [RR, 2.84 (95% CI, 1.63–4.96)] (33). We also performed a comparison of low-dose and standard-dose alteplase in patients with CM-AIS. Eight patients were reported to receive low-dose alteplase. They had a higher incidence of HT and a lower rate of neurological improvement (37.5, 37.5%) than patients who received standard-dose alteplase in our case series (35.7, 64.3%) and pooled analysis (20.5 59.0%). Although the incidence of sICH and the proportion of patients with an mRS ≤ 2 are similar between different dosages of alteplase, low-dose alteplase is not recommended in patients with CM-AIS based on the available evidence.

**Figure 2:**
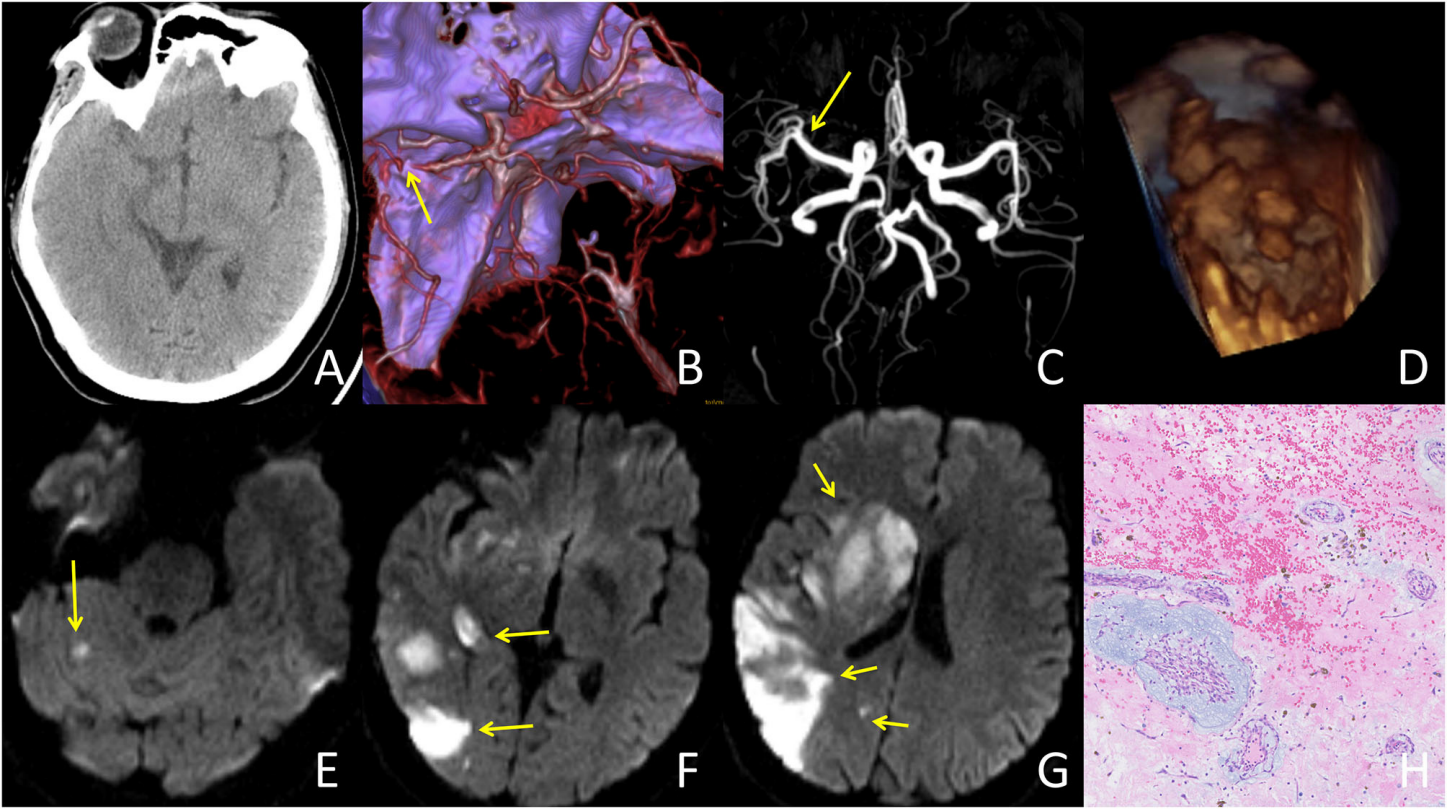
A typical case responded well to IVT treatment: A 42-year-old female presented with an initial NIHSS score of 13. The patient's condition improved dramatically following IVT treatment. **(A)** CT at admission showed no abnormalities. **(B)** CT angiography at admission showed occlusion in the distal M1 segment of the right middle cerebral artery (yellow arrow). **(C)** MR angiography after IVT showed complete recanalization of the right middle cerebral artery. **(D)** TTE showed a left atrial mass with an irregular surface. **(E–G)** DWI demonstrated multiple vascular infarcts, including the right basal ganglia, occipital lobe, temporal lobe, and right cerebellum. **(H)** Histopathologic examination (histopathologic examination (HE) 100×) was consistent with left atrial myxoma with lots of superimposed thrombus.

**Figure 3:**
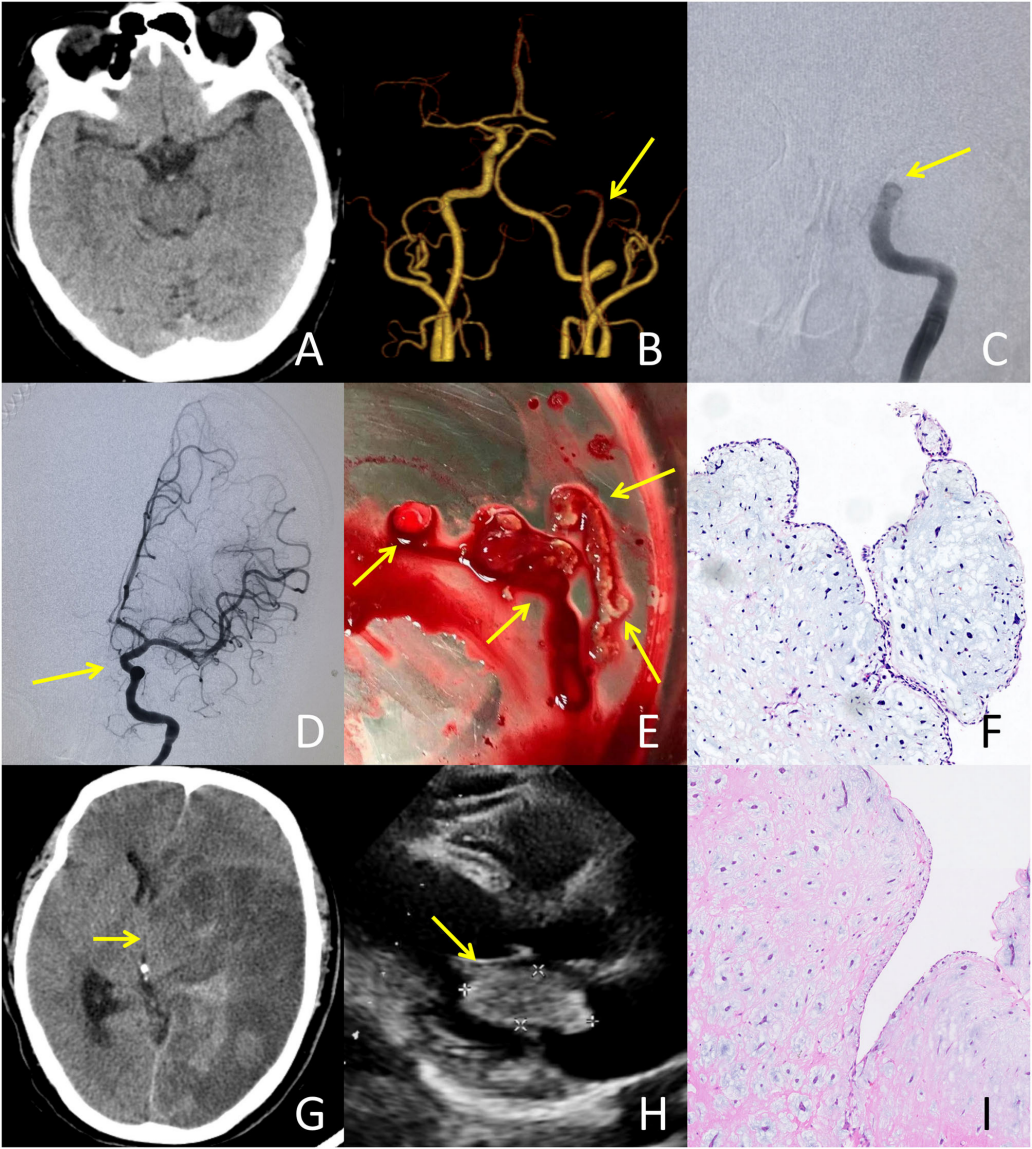
A typical case failed to respond to IVT treatment: A 51-year-old male presented with an initial NIHSS score of 26. A bridging treatment was performed due to the absence of very early neurological improvement. **(A)** CT at admission showed no abnormalities. **(B)** CTA at admission revealed the occlusion of the left internal carotid artery (ICA). **(C)** Digital subtraction angiography after IVT indicated no recanalization of the occlusion in the left ICA occurred. **(D)** Following mechanical thrombectomy, successful recanalization of the left ICA was achieved with a thrombolysis in cerebral infarction (TICI) grade of 3. **(E)** The embolus retrieved from the left ICA exhibited a translucent jelly-like appearance. **(F)** Histopathological examination of the retrieved embolus demonstrated a tumor embolus originating from the cardiac myxoma. There was no thrombus formation within the tissue. **(G)** CT after thrombectomy showed malignant brain edema and hemorrhagic transformation resulting in cerebral herniation, for which the patient underwent decompressive craniectomy. **(H)** Transthoracic echocardiography illustrated a cardiac mass (43 × 20 mm) attached to the left atrial septum. **(I)** Histopathological examination of the cardiac mass showed spindle cells in a fibromyxoid matrix, establishing the diagnosis of cardiac myxoma.

It is well established that VENI is a clinical surrogate of early recanalization (8). The lack of VENI may help clinicians to determine which patients are suitable for BT. In our case series, only one patient had VENI (1/14, 7.1%), and only 10 patients (21.3%) had VENI in the pooled analysis. The incidence of VENI in CM-AIS patients is relatively lower than that in the overall population (30–35.8%) (8). This suggests that IVT may have a smaller effect on early recanalization in CM-AIS patients than in the overall stroke population. The emboli in CM-AIS patients may be part of the CM, a thrombus on the surface of the CM, or a mixture of both (15). However, IVT may only be effective for breaking down the components of the thrombus (Figure 2 demonstrates a typical case that responded well to IVT). In our cases, the retrieved emboli from the three patients who had no VENI were determined to be part of the CM (Figure 3 demonstrates a typical case who was unresponsive to IVT). In a previous review of CM-AIS patients who underwent MT, IVT was performed in 72.7% of patients, but none showed VENI (32), and most of the retrieved emboli were identified as fragments of myxoma. These findings demonstrate that IVT is ineffective for AIS caused by the embolization of a myxoma fragment. Therefore, if VENI is not achieved, BT should be initiated immediately.

Clinical reports of direct MT and BT for CM-AIS patients with LVO have been published (32); moreover, we have administered BT to five patients. MT has several advantages, which include a high recanalization rate, low bleeding risk, and the ability to determine stroke etiology *via* histopathological analysis of retrieved emboli. Furthermore, MT appears to be a feasible, effective, and beneficial therapeutic option, or at minimum a rescue therapy for CM-AIS patients. MT may be superior to IVT for AIS caused by embolization of a myxoma fragment. However, IVT is still recommended as first-line treatment of AIS based on recent RCT evidence demonstrating that BT offers more benefits than MT alone for functional outcomes and recanalization rates without compromising safety (37). Thus, for now, IVT should not be withheld in CM-AIS patients with LVOs. Further studies in a larger cohort may help evaluate the superiority of reperfusion strategies for the treatment of this rare condition.

Recent evidence recommends that surgical resection of CM should be performed as early as possible if the clinical condition is applicable, especially in patients with papillary type myxomas (10, 11, 15, 23). This may be the only option for the prevention of embolism events and improvement of systemic symptoms. There is a range of evidence supporting early surgical resection. Firstly, patients with CM are at a high risk of embolism, intracardiac obstruction, and even sudden death. Patients who undergo CM resection have been reported to have significantly lower mortality (2.1%) than those who do not undergo resection (44.4%) (15). Secondly, both anticoagulants and antiplatelet agents are ineffective in preventing embolism while awaiting surgery (11). Moreover, antithrombotic therapy should not be considered an alternative to surgical resection. In a recent retrospective study, cerebral embolism occurred in nearly half of patients who received previous pharmacological treatment. In addition, cerebral embolism recurrence was observed in 23% of patients who underwent bridging-antithrombotic therapy during the interval between AIS onset and CM surgery (11). In line with this finding, four (28.6%) of our case series patients had thromboembolic events during the bridging interval for surgical excision. Thirdly, prompt CM resection is considered safe, with an operative mortality rate of 0–3% (38). Furthermore, previous reports have suggested that perioperative complications are rare for CM resection (38). However, the optimal timing for surgery after CM-AIS remains controversial. Systemic anticoagulation during surgery may increase the risk of HT in patients who have previously undergone IVT; therefore, determining the time of surgery should follow a multidisciplinary and personalized approach. However, HT was not observed in any of the patients who underwent early resection in a previous study (11). Moreover, mortality does not differ between patients who undergo early resection and those who undergo delayed resection (15). A prolonged interval between AIS and surgical resection is strongly correlated with the recurrence of thromboembolic events (11). Taken together, the above evidence suggests that early surgical resection is imperative, feasible, and safe for the treatment of CM, as recommended by numerous researchers (10, 11, 13, 15).

## Limitations

We acknowledge several limitations to the present study. The first and most notable limitations are related to the small sample size, the retrospective study design, and the selection of reported cases. The sample size of our study was extremely small despite multicentre efforts to identify as many patients with CM-AIS as possible; thus, the overall power and reliability of the study are low. This limits the generalizability of our results to routine clinical practice. Secondly, one limitation is that there was not a non-IVT group with which to make comparisons to determine the impact of IVT treatment; However, only six CM-AIS patients within time-window of reperfusion treatment were identified to be not treated with IVT. Comparisons were unavailable because of the limited sample size. We intend to do this type of comparison in future studies. Thirdly, we did not find any RCTs on the use of IVT for CM-AIS patients. Thus, our systematic review only included case reports and small case series. Moreover, avoiding biases was challenging, which included publication, selection, citation, and heterogeneity biases. Additionally, some important data were missing in the included publications, which made comparisons of results difficult. However, the rarity of CM-AIS limits the conduct of prospective studies, especially those that explore the use of IVT in this particular population. Thus, a systematic review of case reports and case series for rare diseases is warranted.

## Conclusions

To the best of our knowledge, we presented the largest case series of CM-AIS patients treated with IVT. We also performed the most extensive systematic review focused on the use of IVT to treat CM-AIS patients. Our case series and pooled analysis of available literature demonstrates that the use of IVT in CM-AIS patients remains uncommon but appears to be safe, although its efficacy remains to be established. Cerebral angiography is recommended prior to IVT to exclude myxomatous aneurysms and minimize the risk of ICH. In addition, BT should be immediately initiated if VENI is not achieved. Further studies with larger sample sizes are required to provide more evidence on the safety and efficacy of IVT as well as BT for the treatment of CM-AIS patients.

## Data Availability Statement

The original contributions presented in the study are included in the article/Supplementary Material, further inquiries can be directed to the corresponding authors.

## Ethics Statement

Written informed consent was obtained from the individual(s) for the publication of any potentially identifiable images or data included in this article.

## Author Contributions

FF and XC contributed to the design of the study, take responsibility for the integrity, and accuracy of the data. JR and ZT contributed to the acquisition and analysis of the clinical data, drafting the manuscript, and reviewing the published literature. EZ, QB, and MJ contributed to clinical data collection and follow-up. All authors read and approved the final manuscript to be published.

## Funding

This work was supported by the Medical Science and Technology Project of Zhejiang Province of China (No. 2021KY797) and the Public Welfare Technology Applied Research Project of Lishui City (No. 2020SJZC055).

## Conflict of Interest

The authors declare that the research was conducted in the absence of any commercial or financial relationships that could be construed as a potential conflict of interest.

## Publisher's Note

All claims expressed in this article are solely those of the authors and do not necessarily represent those of their affiliated organizations, or those of the publisher, the editors and the reviewers. Any product that may be evaluated in this article, or claim that may be made by its manufacturer, is not guaranteed or endorsed by the publisher.
