# Supplementary material for: Intravenous Thrombolysis for Acute Ischemic Stroke in Patients With Cardiac Myxoma: A Case Series and Pooled Analysis
Source: Front Neurol. 2022 May 12;13:893807. doi: 10.3389/fneur.2022.893807 (PMC9133336; doi:10.3389/fneur.2022.893807)
Supplement: Supplementary file 1 [file Table_1.PDF]

Supplementary Table 1: Results of the systematic literature review of reports on patients with cardiac myxoma-related acute ischemic stroke treated with intravenous thrombolysis.

Author/ Year	Age/ Sex	Risk factor	Initial NIHSS	HDA	LVO	Multivessel territories involvement	Peripheral vascular involvement	Intravenous thrombolysis								BT	Cardiac myxoma						Favorable outcome
								OTN (min)	Dose (mg/Kg)	VENI	NIHSS at 24h	HT	sICH	MBE	mRS at 3 months		Site and Diameter (mm)	Irregular surface	Prestroke symptom	Removal time	Drug before removal	Relapse before removal	
Chong, 2005 (1)	74/F	No	6	No	No	Yes	No	180	0.9	No	NA	Yes, SAH and PH2	Yes	No	3	No	LA, 40	NA	TIA	7 d	NA	No	No
Liao, 2006 (2)	47/M	Smoking	18	No	Left M1	No	No	180	0.9	No	3	Yes, HI1	No	No	0	No	LA, NA	NA	Cerebral infarction	NA	NA	No	Yes
Ibrahim, 2008 (3)	51/M	No	22	No	NA	NA	No	84	0.9	No	NA	No	No	No	0	No	LA, 47; RA, 26	No	No	NA	NA	No	Yes
Lin, 2009 (4)	65/M	HTN, COPD, smoking	20	No	NA	NA	No	NA	0.7	Yes	NA	No	No	No	2	No	LA, 56	Yes	No	7 d	NA	No	Yes
Nagy, 2009 (5)	26/M	No	10	No	No	No	No	105	0.9	Yes	2	No	No	No	1	No	LA, 64	Yes	Syncope	2 d	NA	No	Yes
Nishimura, 2010 (6)	72/M	No	17	No	Right ICA, Right M1	No	No	100	0.6	No	17	No	No	No	5	No	LA, 54	Yes	No	36 d	NA	No	No
Ong, 2010 (7)	22/F	No	12	No	Right M1	Yes	No	125	0.6	No	11	No	No	No	2	No	LV, 40	No	Dyspnea, syncope	NA	NA	No	Yes
Abe, 2011 (8)	70/M	No	11	No	Left M1	No	No	92	0.6	Yes	0	No	No	No	0	No	LA, 70	No	No	20 d	Warfarin	No	Yes
Acampa, 2011	63/F	HTN, HLP	19	No	Right M1	No	No	160	0.9	No	19	Yes, HI2	No	No	4	No	LA, 60	No	No	30 d	Aspirin	No	No

(9)																							
Sun, 2011 (10)	45/M	No	16	No	NA	No	No	172	0.9	No	16	No	No	No	3	No	LA, 80	Yes	No	4 d	NA	No	No
Kohno, 2012 (11)	79/M	HTN	24	No	Left MCA	No	No	180	0.6	No	25	Yes, HI1	No	No	4	No	LA, 21	NA	No	Not performed	Warfarin	No	No
Sudhakar, 2012 (12)	64/M	HTN, CHD	NA	No	NA	NA	No	NA	0.9	No	NA	No	No	No	2	No	LV, 25	No	Cardiac arrest, ventricular fibrillation	3 d	NA	No	Yes
Silva, 2012 (13)	69/F	HTN	12	NA	Right MCA	No	Left femoral artery	90	0.9	Yes	5	No	No	Yes	6	No	LA, 20	NA	TIA	2 d	Aspirin, enoxaparin	left femoral artery embolism	No
Hatayama, 2012 (14)	76/M	HTN, DM	17	No	No	Yes	No	155	0.6	No	14	Yes, HI1	No	No	3	No	LA, 35	Yes	No	33 d	Heparin	Cerebral infarction	No
Ružička-Kaloci, 2012 (15)	42/F	No	17	No	No	No	No	NA	0.9	No	NA	No	No	No	2	No	LA, 45	Yes	No	1 d	NA	No	Yes
Alsindi, 2012 (16)	49/F	No	11	No	NA	No	No	NA	0.9	No	NA	No	No	No	2	No	LA, 45	No	No	NA	NA	No	Yes
Habbab, 2014 (17)	52/F	Smoking, CHD	NA	Yes	Right ICA	No	Infrarenal abdominal aorta, iliacs, and superficial femoral arteries	NA	0.9	No	NA	Yes, HI2	No	Yes	5	No	LA, 48	NA	Shortness of breath	Not performed	NA	No	No
Kulkarni, 2014(18)	69/F	HTN,DM	14	No	No	No	No	NA	0.9	Yes	31	Yes, PH2	Yes	No	4	No	LA, 27	Yes	No	Not performed	NA	No	No

Chutinet, 2014(19)	58/M	HTN	20	No	No	Yes	No	140	0.9	No	22	Yes, PH2	Yes	Yes	4	No	LA, 40	Yes	Cerebral infarction	NA	NA	No	No
IsmailII, 2015(20)	42/F	No	21	Yes	Right M1	No	No	210	0.9	Yes	15	No	No	No	4	No	LA, 80	NA	Cough, palpitation and chest tightness	Not performed	Warfarin	No	No
Jia,2016 (21)	53/M	No	14	No	NA	No	No	NA	0.9	No	NA	No	No	No	3	No	LA, 74	NA	No	NA	NA	No	No
Ikeda, 2016 (22)	74/M	HTN, DM, LEASO	7	No	No	Yes	Left upper extremity artery	200	0.9	No	4	No	No	No	2	No	LA, 30	Yes	Cerebral infarction	14 d	Clopidogrel	No	Yes
Dai,2016 (23)	58/F	No	6	No	No	No	No	109	0.9	No	NA	No	No	No	2	No	LA, 66	No	No	7 d	Aspirin	No	Yes
Rao,2016 (24)	62/F	No	2	NA	NA	No	No	NA	0.9	No	NA	No	No	No	0	No	RV, 51	Yes	No	21 d	NA	TIA	Yes
Vidale,2017 (25)	62/F	HTN	18	No	No	No	No	180	0.9	Yes	6	No	No	No	2	No	LV, 60	No	No	4 d	NA	No	Yes
Lee,2017 (26)	77/F	HTN	18	No	NA	NA	Bilateral lower pulmonary arteries	NA	0.9	No	NA	No	No	No	3	No	LA, 46.6 RA, NA	Yes	No	NA	NA	Pulmonary embolism	No
Díaz,2018 (27)	60/F	DM, HLP, COPD, Smoking	NA	No	No	Yes	No	180	0.9	Yes	NA	No	No	No	1	No	LA, NA	Yes	No	15 d	Anticoagul-ants	No	Yes
Han,2018 (28)	57/F	No	4	No	No	Yes	No	NA	0.9	Yes	0	No	No	No	0	No	LA, NA	NA	No	7 d	NA	No	Yes
Alkuwaiti, 2018 (29)	40/M	No	NA	No	NA	Yes	No	NA	0.9	No	NA	No	No	No	0	No	AV, 6	NA	Stroke	14 d	Aspirin, clopidogrel	No	Yes
Kim,2018 (30)	48/M	Smoking	17	No	No	No	No	65	0.9	No	NA	No	No	No	1	No	LA, 30	Yes	No	14 d	NA	No	Yes

Zhang,2020 (31)	53/M	No	15	No	No	Yes	No	90	0.9	No	NA	No	No	No	3	No	LA, 36	Yes	No	Not performed	Aspirin	No	No
Esmaeili, 2020 (32)	31/M	No	10	No	NA	No	No	170	0.9	No	1	No	No	No	0	No	LA, 50	Yes	No	90 h	Heparin	No	Yes
Garcia-Ptacek, 2014 (33)	45/NA	No	22	No	Left M1	Yes	Bilateral iliac and distal aortic thrombosis	100	0.9	No	NA	No	No	No	4	Yes	LA, NA	NA	No	NA	NA	No	No
Garcia-Ptacek, 2014 (33)	34/NA	No	26	No	Left M1, Left A2	Yes	No	190	0.9	No	NA	No	No	No	2	Yes	LA, NA	NA	No	NA	NA	No	Yes
Kamiya, 2014 (34)	48/F	No	32	No	BA, Right M2	Yes	No	120	0.9	No	NA	No	No	No	5	Yes	LA, 54	Yes	No	20 d	Warfarin	No	No
Baek, 2014 (35)	46/M	No	NA	No	Left distal ICA, Left M2	No	No	NA	0.9	No	NA	No	No	No	1	Yes	LA, 42	Yes	No	5 d	NA	Left cilioretinal artery occlusion	Yes
van den Wijngaard, 2014(36)	14/M	No	21	No	Left M1	Yes	No	240	0.9	No	NA	No	No	No	2	Yes	LA, NA	NA	Typical skin lesions	1 d	NA	Left central retinal artery occlusion	Yes
Ryu, 2015 (37)	34/M	Smoking	9	Yes	Right M1	No	No	113	0.6	No	6	Yes, NA	No	No	2	Yes	LA, 14	NA	No	1 m	Warfarin	No	Yes
Uneda, 2016 (38)	70/M	No	11	NA	Left T-ICA, Left M2	Yes	No	175	0.9	No	NA	No	No	No	2	Yes	LA, 40	NA	No	6 d	NA	No	Yes
Chung, 2016 (39)	4/M	No	16	No	Left M1	Yes	No	210	0.6	No	NA	No	No	No	1	Yes	LA, 20	NA	Febrile convulsion	7 d	NA	No	Yes
Zapata-Arri	17/M	No	32	No	Right ICA,	Yes	Left	95	0.9	Yes	NA	Yes,	Yes	Yes	6	Yes	LA,	Yes	No	Not	NA	No	No

aza, 2017(40)					Right M1, Left M1-M2, Right PCA		subclavian artery, abdominal aorta, both iliac and femoral arteries					PH2					NA			performed			
Kuwahara, 2018(41)	31/M	No	9	No	Right M2	No	No	87	0.9	No	NA	No	No	No	0	Yes	LA, NA	NA	No	6 d	NA	No	Yes
Tadi, 2019 (42)	23/F	Smoking, obesity, oral contracept-ive	19	No	Left M1	No	No	NA	0.9	No	10	No	No	No	2	Yes	LA, 70	Yes	No	8d	Aspirin	Cerebral infarction	Yes
Cai, 2019 (43)	61/M	No	16	No	Left M1	No	No	NA	0.9	No	5	No	No	No	0	Yes	LA, 45	NA	No	21 d	Aspirin, clopidogrel	No	Yes
Li, 2019 (44)	61/M	No	14	No	Left M1	No	Left external carotid artery	NA	0.9	No	NA	No	No	No	2	Yes	LA, 32	NA	No	30 d	NA	No	Yes
Zhang, 2020 (45)	15/F	No	26	Yes	Left M1	No	No	60	0.9	No	NA	Yes, SAH and PH2	No	No	3	Yes	LA, 35	NA	No	Not performed	Antiplatelet	No	No
Chang, 2021 (46)	42/F	No	18	No	Left M1	No	No	140	0.9	No	3	No	No	No	0	Yes	LA, 65	Yes	No	2 d	NA	No	Yes

A2=second segment of anterior cerebral artery; AV=aortic valve; BA=basilar artery; BT=bridging therapy; CHD=coronary heart disease; COPD=chronic obstructive pulmonary disease; DM=diabetes mellitus; HDA=high density sign of artery; F=female; HI=hemorrhagic infarction; HLP=hyperlipidaemia; HT=hemorrhagic transformation; HTN=hypertension; ICA=internal carotid artery; LA=left atrium; LEASO=lower extremity arteriosclerosis obliterans; LV=left ventricular; LVO=large vessel occlusion; M=male; M1=proximal segment of middle cerebral artery; M2=second segment of middle cerebral artery; MBE=malignant brain edema; MCA=middle cerebral artery; NA=not available. NIHSS=National Institutes of Health Stroke Scale; mRS=modified Rankin Scale; OTN=Onset-to-needle time; PCA=posterior

cerebral artery; PH=parenchymal hemorrhage; RA=right atrial; RV=right ventricular; SAH=subarachnoid hemorrhage; sICH=symptomatic intracerebral hemorrhage; TIA=transient ischemic attack; VENI=very early neurological improvement;

REFERENCES

1. Chong JY, Vraniak P, Etienne M, Sherman D, Elkind MS. Intravenous thrombolytic treatment of acute ischemic stroke associated with left atrial myxoma: a case report. J Stroke Cerebrovasc Dis. 2005;14(1):39-41.
2. Liao X, Wang Y, Wang Y, Dong K. A case of successfully treated with Intravenous fibrinolysis for acute cerebral embolism caused by atrial myxoma. Chinese Journal of Nervous and Mental Diseases. 2006;32(05):470-1.
3. Ibrahim M, Iliescu C, Safi HJ, Buja ML, McPherson DD, Fuentes F. Biatrial myxoma and cerebral ischemia successfully treated with intravenous thrombolytic therapy and surgical resection. Tex Heart Inst J. 2008;35(2):193-5.
4. Lin P, Wu H, Yu S, Ke CJTCCM. Acute stroke and left atrial myxoma successfully treated with intravenous thrombolytic therapy and surgical resection-case report. 2009;10:333-40.
5. Nagy CD, Levy M, Mulhearn TJt, Shapland M, Sun H, Yuh DD, et al. Safe and effective intravenous thrombolysis for acute ischemic stroke caused by left atrial myxoma. J Stroke Cerebrovasc Dis. 2009;18(5):398-402.
6. Nishimura H, Nakajima T, Ukita T, Tsuji M, Miyake H, Ohmura T, et al. A case of acute cerebral infarction associated with left atrial myxoma treated by intravenous tissue plasminogen activator. 2010;32:156-62.
7. Ong CT, Chang RY. Intravenous thrombolysis of occlusion in the middle cerebral and retinal arteries from presumed ventricular myxoma. Stroke Res Treat. 2010;2011:735057.
8. Abe M, Kohama A, Takeda T, Ishikawa A, Yamada Y, Kawase Y, et al. Effective intravenous thrombolytic therapy in a patient with cerebral infarction associated with left atrial myxoma. Intern Med. 2011;50(20):2401-5.
9. Acampa M, Tassi R, Guideri F, Marotta G, Monti L, Capannini G, et al. Safety of intravenous thrombolysis in ischemic stroke caused by left atrial myxoma. 2011;6(5):343-5.
10. Sun MC, Tai HC, Lee CH. Intravenous Thrombolysis for Embolic Stroke due to Cardiac Myxoma. Case Rep Neurol. 2011;3:21-6.
11. Kohno N, Kawakami Y, Hamada C, Toyoda G, Bokura H, Yamaguchi S. Cerebral embolism associated with left atrial myxoma that was treated with thrombolytic therapy. Case Rep Neurol. 2012;4(1):38-42.
12. Sudhakar S, Robinson P, Loyo J, Hai H, Sewani A. An unusual case of left ventricular myxoma. Journal of cardiovascular medicine (Hagerstown, Md). 2012;13(6):410-2.
13. da Silva IR, de Freitas GR. Is it safe to proceed with thrombolytic therapy for acute ischemic stroke in a patient with cardiac myxoma? Case report and review of the literature. Eur Neurol. 2012;68(3):185-6.
14. Hatayama S, Ogata T, Okawa M, Higashi T, Inoue T, Takano K, et al. [Ischemic stroke induced by a left atrial myxoma]. Brain Nerve. 2012;64(10):1175-9.
15. Ruzicka-Kaloci S, Slankamenac P, Vitic B, Lucic-Prokin A, Jovicevic M, Zivanovic Z, et al. Atrial myxoma as a cause of stroke: emboli detection and thrombolytic treatment. Med Glas (Zenica). 2012;9(1):114-7.
16. Alsindi F, Duggirala V, Villanueva AJC. Successful thrombolysis and excision of left atrial myxoma presenting with weakness. 2012;142(4):122A.
17. Habbab L, Alfaraidi H, Lamy A. Surviving catastrophic disintegration of a large left atrial myxoma: the importance of multi-disciplinary team. J Surg Case Rep. 2014;2014(9).
18. Kulkarni GB, Yadav R, Mustare V, Modi S. Intravenous thrombolysis in a patient with left atrial myxoma with acute ischemic stroke. Annals of Indian Academy of Neurology. 2014;17(4):455-8.
19. Chutinet A, Roongpiboonsopit D, Suwanwela NC. Intracerebral hemorrhage after intravenous thrombolysis in patients with cerebral microbleeds and cardiac myxoma. Front Neurol. 2014;5:252.
20. Ismail I, Salama S, Mansour OJJCRS. A Case Report of Atrial Myxoma presenting with Cardioembolic Stroke and treated with Intravenous Thrombolytic Therapy. 2015;3(2):205.
21. Jia L, Jing Y, Zhao Y. A case of acute cerebral embolism caused by left atrial myxoma treated with intravenous alteplase. Translational Medicine Journal. 2016;5(01):62-4.
22. Ikeda T, Oomura M, Sato C, Anan C, Yamada K, Kamimoto K. Cerebral infarction due to cardiac myxoma developed with the loss of consciousness immediately after defecation-a case report. Rinsho Shinkeigaku. 2016;56(5):328-33.
23. Dai M, Xu D, Chen J, Zhang W. A case of acute Ischemic Stroke caused by left atrial myxoma. Zhejiang Practical Medicine. 2016;21(04):309-10.
24. Rao PA, Nagendra Prakash SN, Vasudev S, Girish M, Srinivas A, Guru Prasad HP, et al. A rare case of right ventricular myxoma causing recurrent stroke. Indian Heart J. 2016;68 Suppl 2:S97-S101.

25. Vidale S, Comolli F, Tancredi L, Campana C, Arnaboldi M. Intravenous thrombolysis in a patient with left atrial myxoma. Neurol Sci. 2017;38(7):1345-6.
26. Lee WC, Chen HC, Chua S. Systemic embolism from bilateral atrial myxomas. J Echocardiogr. 2018;16(2):89-90.
27. Diaz Diaz A, Garcia AM, Sedeno GP, Garcia Rodriguez JR. Intravenous fibrinolysis for acute ischaemic stroke associated with left atrial myxoma: A case report. Neurologia (Engl Ed). 2018;33(4):267-8.
28. Han B. A case of thrombolysis for basilar artery infarction caused by atrial myxoma. China Rural Health. 2018(10):83.
29. Alkuwaiti FA, Elghoneimy Y, Ghazal S. Aortic Valve Myxoma Presenting with a Stroke: A case report and review of the literature. Sultan Qaboos Univ Med J. 2018;18(4):e537-e40.
30. Kim EY, Jeong HY, Lee YSJJotKNA. Intravenous Thrombolysis Use in Acute Stroke with Cardiac Myxoma. 2018;36(4):408-10.
31. Zhang H, Xie Z, Che F. A case report of thrombolysis for ultra-acute cerebral embolism associated with atrial myxoma. Chinese Journal of Stroke. 2020;15(01):89-92.
32. Esmaeili S, Shojaei SF, Bahadori M, Mojtahed M, Mehrpour M. Intravenous Thrombolysis for Acute Ischemic Stroke Due to Cardiac Myxoma. Basic Clin Neurosci. 2020;11(6):855-9.
33. Garcia-Ptacek S, Matias-Guiu JA, Valencia-Sanchez C, Gil A, Bernal-Becerra I, De las Heras-Revilla V, et al. Mechanical endovascular treatment of acute stroke due to cardiac myxoma. J Neurointerv Surg. 2014;6(1):e1.
34. Kamiya Y, Ichikawa H, Mizuma K, Itaya K, Shimizu Y, Kawamura M. [Case of acute ischemic stroke due to cardiac myxoma treated by intravenous thrombolysis and endovascular therapy]. Rinsho Shinkeigaku. 2014;54(6):502-6.
35. Baek SH, Park S, Lee NJ, Kang Y, Cho KH. Effective mechanical thrombectomy in a patient with hyperacute ischemic stroke associated with cardiac myxoma. J Stroke Cerebrovasc Dis. 2014;23(9):e417-9.
36. van den Wijngaard I, Wermer M, van Walderveen M, Wiendels N, Peeters-Scholte C, Lycklama ANG. Intra-arterial treatment in a child with embolic stroke due to atrial myxoma. Interv Neuroradiol. 2014;20(3):345-51.
37. Ryu B, Ishikawa T, Sato S, Yokote A, Nakamoto H, Nie M, et al. Mechanical Endovascular Recanalization in a Patient with Middle Cerebral Artery Occlusion by Tumorous Emboli Originating from Cardiac Myxoma. NMC Case Rep J. 2015;2(2):53-6.
38. Uneda A, Suzuki K, Hirashita K, Yoshino K. Tandem cervical/intracranial internal carotid artery occlusion due to cardiac myxoma treated successfully with mechanical endovascular thrombectomy. Acta Neurochir (Wien). 2016;158(7):1393-5.
39. Chung YS, Lee WJ, Hong J, Byun JS, Kim JK, Chae SA. Mechanical thrombectomy in cardiac myxoma stroke: a case report and review of the literature. Acta Neurochir (Wien). 2016;158(6):1083-8.
40. Zapata-Arriaza E, Pardo-Galiana B, Gonzalez-Garcia A, Montaner Villalonga J. Intravenous thrombolysis and thrombectomy in young patients with ischaemic stroke due to undetected atrial myxoma: Do recent clinical trials provide sufficient evidence to support reperfusion in these cases? Neurologia. 2017;32(6):404-7.
41. Kuwahara K, Moriya S, Maeda S, Hayakawa M, Mizoguchi Y, Nakahara I, et al. Cardiogenic Embolism due to Left Atrial Myxoma Successfully Treated by ADAPT: A Case Report. 2017;12(4):193-8.
42. Tadi P, Feroze R, Reddy P, Sravanthi P, Fakhri N, McTaggart R, et al. Clinical Reasoning: Mechanical thrombectomy for acute ischemic stroke in the setting of atrial myxoma. Neurology. 2019;93(16):e1572-e6.
43. Cai X, Xu H, Liu J, Dai Y, He W, Li J, et al. Endovascular thrombectomy after intravenous recombinant tissue plasminogen activator (bridging therapy) for embolic stroke due to cardiac myxoma: a case report. Chinese Journal of Neurology. 2020;53(2):118-21.
44. Li J, Tang X, Cai X, Wang K, Yan J. Mechanical thrombectomy for middle cerebral artery embolization caused by atrial myxoma: a case report and literature revie. Chinese Interventional Neurology Meeting 2019-The 15th International Stroke Summit. 2019:88-9.
45. Zhang P, Hu F, Sun Y, Niu W, Wang J, Guo W. One case report of intravenous thrombolysis bridging endovascular thrombectomy for ischemic stroke caused by juvenile atrial myxoma. Chinese Journal of Nervous and Mental Diseases. 2020;3:166-8.
46. Chang WS, Li N, Liu H, Yin JJ, Zhang HQ. Thrombolysis and embolectomy in treatment of acute stroke as a bridge to open-heart resection of giant cardiac myxoma: A case report. World J Clin Cases. 2021;9(25):7572-8.
